# Global Initiative for Children’s Surgery (GICS): a decade in review

**DOI:** 10.1136/wjps-2025-001084

**Published:** 2025-10-02

**Authors:** Soham Bandyopadhyay, Godfrey Sama Philipo, Tahmina Banu, Zaitun Bokhary, Kokila Lakhoo

**Affiliations:** 1 Global Surgery Group, Nuffield Department of Surgical Sciences, Oxford University, Oxford, Oxfordshire, UK; 2 Muhimbili National Hospital, Dar es Salaam, Tanzania; 3 Chittagong Medical College Hospital, Chattogram, Chittagong Division, Bangladesh

**Keywords:** Child Health, Education, Medical, Evidence-Based Medicine, Health Services, Schools, Public Health

## Origins and principles

 As progress in combating infectious diseases has led to more children surviving and morbidity reducing, surgical conditions have accounted for a larger share of global ill health.^1^ Yet, 5 billion people lack safe, affordable operative care^2^; approximately 50% of them are children.^3^ Without decisive action, the Sustainable Development Goals (SDGs) for children’s health will remain out of reach.^4^ Preventable deaths and life-long disability in childhood can also push families into poverty and sap national productivity.^5^ Absent, unaffordable, or poor-quality surgical care for children is therefore both a moral and an economic crisis.^6 7^

Children in low- and middle-income countries (LMICs) bear the brunt; in some of the poorest regions, they make up roughly half the population, but only a fraction have access to the surgical services they require.^8 9^ As an example, over 20 million Ugandan children depend on just three pediatric surgeons and three pediatric anesthetists: a ratio so low that many urgent cases are never seen, let alone treated.^10 11^

Historically, surgery was dismissed as the “neglected step-child of global health”, absent from policy discourse until the mid-2010s. Momentum shifted in 2015 “the year of global surgery”—when the Lancet Commission revealed the unmet need and urged countries to weave surgery into universal health coverage.^2^ Yet even that landmark report dealt mainly with adult services; the specific requirements of children were left largely unaddressed.^8 12^ The story that follows explains how we have begun to close this gap.

Children’s surgery is not simply “smaller” adult surgery: children need differently sized equipment, teams trained in distinct physiology and pathology, and services that span congenital anomalies as well as injuries and tumors.^13 14^ Yet when governments began drafting National Surgical, Obstetric and Anaesthesia Plans (NSOAPs) after the 2015 Lancet Commission, pediatric needs were again in danger of slipping through the cracks.^15^ To close that gap, the Global Initiative for Children’s Surgery (GICS) was launched in May 2016 by pediatric surgeons, anesthetists, nurses and child-health advocates from across income settings.^16 17^ From the outset, the initiative set three rules: be inclusive, be equitable, and let the people who deliver care in low-resource settings lead the agenda. In contrast with many top-down global health campaigns, GICS is a grassroots coalition of frontline clinicians—most of them based in LMICs—who design the solutions they themselves will use. GICS also looks beyond hospitals and ministries to the people most affected by surgical gaps: children and their families. Parent groups for conditions such as spina bifida or clubfoot are being invited into planning meetings and advocacy campaigns, adding lived experience to the data-driven case for investment. This shift toward community voice reflects a rights-based view of health, one that treats access to children’s surgery as a fundamental entitlement rather than a discretionary service.

The inaugural meeting in London gathered 52 delegates from 21 countries with more than half from LMICs. They agreed that every child should have timely, safe and affordable surgical, anesthetic and nursing care, and identified four pillars for progress: infrastructure, service delivery, training and research.^18^ Early priorities were embedding children’s surgery within NSOAPs, ensuring there was at least one children’s hospital in every country, creating regional training hubs, and establishing research support centers to generate local evidence.^19^

A small “core team” was created to keep momentum between biennial conferences by coordinating communications, supporting studies, organizing virtual meetings and amplifying GICS on social media. Day-to-day work is carried out through a matrix of cross-cutting committees spanning specialties, sectors and continents. Clinical working groups cover the breadth of surgical specialities and healthcare professionals required for safe surgical care in childhood.^16^ GICS also maintains committees for training, research, infrastructure, policy, and partnerships. These groups meet regularly (often virtually) to exchange ideas, develop resources, and report back at GICS’s biennial meetings.

## Building standards

Translating vision into action required an agreed yardstick for what safe children’s surgery should look like at every tier of a health system. Between 2016 and 2020, GICS therefore led the Optimal Resources for Children’s Surgery (OReCS) project, a consensus exercise driven chiefly by clinicians from LMICs.^20^,^22^ Drafted at successive meetings in Washington, DC, USA (2016), Vellore, India (2018), Johannesburg, South Africa (2020) and refined through virtual working groups, OReCS sets out in plain, operational terms the human resources, infrastructure, equipment, and supplies required for children’s surgical care at primary, first-level, secondary, and tertiary facilities. The document covered everything from the minimum number and types of trained personnel (surgeons, anesthetists, nurses) at each facility tier, to the availability of pediatric-sized equipment (airway tubes, surgical instruments), essential medications, blood banking, and infrastructure like child-friendly recovery areas.^20^ For instance, OReCS recommends that every country have at least one tertiary children’s hospital and that district hospitals (second level hospitals) be equipped to perform a set of pediatric procedures (such as hernia repairs, appendectomies, treatment of common fractures) with proper pediatric anesthetic support. The guidelines also emphasize systems for safe anesthesia and perioperative care for neonates and children, recognizing children’s unique physiology. Because it was written by those who know the constraints first-hand, the OReCS project is a hallmark of GICS’s approach: LMIC-led consensus on standards, intended for practical use in policy and planning.

## Policy and advocacy impact

Translating a blueprint into better outcomes requires political traction, and influence in the corridors where health budgets and priorities are set. Guided by OReCS, GICS has provided a tool and/or persuaded governments, professional bodies and multilateral agencies that children’s surgery is written into policy documents and prioritized.^23^ Much of this effort has focused on NSOAPs. In Tanzania, the children’s surgery team developed a national roadmap with recommendations slated for inclusion in the 2026 NSOAP renewal.^24^ In Pakistan, a GICS-led situational analysis underpinned the National Vision for Surgical Care,^25^ which explicitly lists children’s surgery as a core UHC component.^16^ In Nigeria, pediatric targets—district-level task-sharing, additional operating theaters, and neonatal critical-care capacity—were written into the 2019–2023 NSOAP; early roll-out has already opened dedicated theaters and reduced hernia/appendicitis waiting lists.^26^

GICS’s advocacy does not stop at national borders. Its representatives remind global child-health forums—from newborn-care meetings to nutrition summits—that many program goals cannot be met while surgical needs go unanswered. They have urged ministries to track children’s surgical volume and postoperative mortality alongside familiar indicators such as immunization coverage. Working with the WHO, GICS updated the WHO’s manual of “Surgical Care at the District Hospital” to include pediatric chapters.^16^ GICS has also fed evidence into UN debates on how surgery advances the SDGs. Birth defects provide a telling case: as infectious-disease deaths fall, congenital anomalies now account for a growing share of under-five mortality in LMICs. In 2024, GICS experts co-authored an international call to scale up corrective surgery for congenital anomalies such as cleft lip and spina bifida, among other surgically correctable birth defects.^27 28^ The working birth defects team in GICS successfully framed these surgical pathologies and other structural birth defects as an essential element of national child health plans. Each such partnership widens the circle of decision-makers who see surgery for children not as a luxury but as a prerequisite for equity.

The cumulative effect is a snowballing network of champions. GICS meetings—already among the largest gatherings of multidisciplinary children’s-surgical advocates—now draw hundreds of clinicians, policy makers and donors who return home with policy templates and an urgency to act. Their collective efforts are beginning to shift children’s surgery from “forgotten cost centre” to “fundamental investment”, laying the political groundwork for their training, infrastructure and research initiatives.^12^

From the outset, GICS has sought out partners whose mandates complement its own, linking standards and policy with on-the-ground delivery.^29^ It works hand-in-glove with other organizations, co-timing conferences and sharing technical working groups. Global health non-governmental organization (NGOs) add further breadth: Smile Train brings expertise in cleft care; Médecins Sans Frontières offers logistics in conflict zones; and Kids Operating Room (KidsOR) installs child-specific theaters across Africa and South America. Even OReCS benefited from outside scrutiny in quality-improvement principles which were woven into its LMIC-led standards, ensuring credibility across income settings.^20^,^22^

## Education and capacity building

That same spirit of collaboration has underpinned GICS’ educational policies. In 2021, GICS clinicians, with support from KidsOR, launched the Pan-African Children’s surgery E-Learning Program (PAPSEP), the first continent-wide curriculum aligned to regional college examinations.^30^ Another example is InterSurgeon. The InterSurgeon web tool—publicized and refined through GICS networks—now matches hospitals that lack expertise or kit with volunteers and donated equipment worldwide, making mentorship and material support far easier to secure.^31^

Partnerships have real-world impact at country level. In Tanzania and Uganda, a GICS-affiliated team convened surgeons, anesthetists, nurses, managers and health officials to tackle chronic bottlenecks. By mapping referral routes, training general surgeons in basic pediatric procedures and opening multidisciplinary children’s wards, they began to unblock a backlog of emergency and elective cases.^17^ This is proof that cross-sector coordination of stakeholders can pay dividends in improving surgical care for children even where specialist numbers remain small.

Building skills is another core tenet of the organization. GICS approaches this on several fronts: supporting training programs, developing educational resources, and fostering south-to-south collaboration in skills transfer. One notable initiative has been the development of low-cost simulation models for pediatric surgical training. Traditional high-fidelity surgical simulators are expensive and seldom available in low-resource settings.^32^ In response, GICS members created a series of low-fidelity pediatric surgical simulators using inexpensive, locally available materials. They designed wet-lab models of common pediatric procedures—neonatal bowel anastomosis, duodenal atresia repair, gastrostomy, and pyeloplasty—constructed from animal tissue and basic instruments.^33^ These models are easily reproducible and provide realistic practice for surgical trainees. The key was to ensure the simulations require only materials and tools readily found in LMIC hospitals (e.g., goat or chicken intestine for bowel surgery practice), making them sustainable. By publishing the methods open-access, GICS enabled training centers worldwide to replicate the models and integrate them into children’s surgery teaching. The outcome is improved surgical skills and confidence among trainees, which ultimately contributes to safer surgery for children.

Another exemplar is GICS emphasis on “south-to-south” exchange as an alternative to costly placements in high-income countries. A case in point is the pediatric surgical training program that was developed in India and transferred to Africa.^34^ This program, piloted in India, focused on training not just surgeons but also anesthetists and nurses as a team to handle common pediatric surgical emergencies at district hospitals. The concept recognized that improving rural surgical access for children requires empowering local general doctors with pediatric surgical skills and providing the necessary equipment and mentoring. By 2024, this idea culminated in a south-to-south initiative where an Indian children’s surgery department helped establish a similar training scheme in Africa. The collaboration involved adapting curricula to the African context and exchange visits for trainers and trainees. The experience demonstrated the feasibility of LMIC-to-LMIC knowledge transfer and its advantages: cultural and resource-context relevance, and the fostering of regional self-reliance. GICS played a facilitating role by connecting the parties and endorsing the program as a model for other regions.

Expanding skills is only half the task; expanding minds matters just as much.^35^ Therefore, GICS has concurrently pressed for a fuller, fairer place for global surgery in university and postgraduate curricula. A 2021 review of existing programs found most were ad hoc offerings written by and for high-income institutions, with scant leadership from LMIC surgeons.^36^ GICS has argued that curricula should be co-designed with low- and middle-income partners, built around two-way exchange, and led by teachers who understand the realities of under-resourced theaters. Its own experience shows how. Grand Rounds and webinars routinely feature children’s surgeons from Africa, Asia and Latin America teaching peers on every continent, reversing the traditional flow of expertise. The same network links young clinicians from LMICs to fellowships and research supervisors, building a pipeline of future leaders who will shape child surgery in their own settings.

## Research and evidence generation

From the outset, GICS also maintained that progress must rest on evidence. It created a Research Committee to coordinate multicenter studies and mentor investigators new to formal inquiry. One of its flagship efforts, the Global PaedSurg Project led by GICS member, collected prospective data on congenital-anomaly outcomes in 74 countries.^37^ Mortality for babies with common gastrointestinal defects was more than 10 times higher in the poorest hospitals than in the richest, but the dataset also highlighted practical levers—neonatal theaters, specialist anesthetists, reliable pulse oximetry—that narrow those gaps. GICS members can now cite the findings when advocating for newborn surgical units and perioperative training. GICS’s role as the evidence engine of global children’s surgery can also be seen in its contribution of “Surgery and the First 8 000 Days of Life” to the Disease Control Priorities series.^13^

GICS has also supported increasing research capacity alongside growing the evidence base. Each GICS meeting includes workshops on study design and data analysis tailored to resource-limited contexts. GICS also created a research-webinar series that paired early-career surgeons with seasoned researchers. The result was a steady rise in first-authored and senior-authored papers from LMIC members, covering topics from task-sharing outcomes to cost–benefit studies of blood-transfusion practice. Open-access routes such as HINARI ensured that the hospitals generating the data could also read and use them.

## Resilience in crises

It has also allowed GICS to dynamically react to changing situations. For example, the COVID-19 pandemic posed unprecedented challenges to surgical care globally, and children’s surgery was no exception. Elective surgeries were delayed, resources were diverted, and training was disrupted.^38^ GICS responded rapidly to support the children’s surgical community during the crisis. Leveraging its international network, GICS organized two online Action Planning Forums in 2020. These forums brought together children’s surgeons and anesthesiologists from across Africa, Asia, the Americas, and Europe to share experiences and develop mitigation strategies.^39^ Discussion ranged from shortages of personal protective equipment to the difficulty of transporting sick newborns across locked-down borders; out of it came pragmatic tools: triage guidance for pediatric emergencies during COVID surges, protocols for safe anesthesia in infected children, and caregiver leaflets to mitigate the harm of delayed presentations.^40 41^ All were collated in an open-access “COVID-19 compendium” on the GICS website, alongside webinars on restarting elective lists, safeguarding trainee education and protecting staff mental health. Crucially, the traffic was two-way: East Asian teams that faced the first wave shared strategies with colleagues in Europe and North America, underscoring GICS’s conviction that expertise flows in every direction.^42^ The episode, therefore, became a live demonstration of global surgical solidarity and of GICS’s ability to pivot from conference podiums to real-time problem-solving when children’s access to surgery is at stake.

## Future directions

As GICS continues to mature, it is branching into specific focal initiatives to address particularly neglected areas within children’s surgery. One such area is pediatric trauma care. Trauma is a leading cause of death and disability in children over 5 years old globally, yet many LMICs lack organized trauma care systems for children.^43^ GICS has been working to develop end-to-end trauma care for children—from prevention to rehabilitation—in resource-limited settings. GICS’s Trauma Group has mapped an “end-to-end” pathway that begins with community first-aid training and runs through child-equipped ambulances, pediatric Advanced Trauma Life Support in district hospitals, age-appropriate operating-theater and intensive care kit, postoperative rehabilitation and injury-prevention programs such as road-safety and burn-awareness campaigns. To seed that model, the group has produced training manuals, piloted a 1-day Children’s Primary Trauma Course at GICS meetings and helped the WHO add a pediatric module to its Emergency Care Systems toolkit.^44^ Advocacy continues in parallel: representatives have raised the issue at the UN General Assembly, arguing that the anatomical, physiological and logistical nuances of childhood injury demand strategies distinct from adult trauma care. Over the coming years, GICS plans to work with pilot countries to implement child-focused trauma improvements, which could serve as models for regional scale-up.

Another growing focus is on neonatal surgical care and the early neonatal period, aligned with the broader newborn health agenda. Conditions like gastroschisis, neonatal sepsis due to surgical conditions, and congenital diaphragmatic hernia have extremely high mortality in low-resource settings.^27 28 45 46^ GICS is working with maternal–newborn health alliances to ensure that surgical care appears in “first 1000 days” policies that cover pregnancy through a child’s second birthday. The agenda includes timely antenatal diagnosis, safe neonatal transfer, newborn-specific anesthesia protocols and family-centered postoperative support. By embedding surgery in the wider continuum of care, GICS aims to show that surviving the first days of life is not only about vaccines and antibiotics but also about having the scalpel, the skills and the staffed incubator ready when a baby is born with a correctable defect.

By staying transparent, inclusive and relentlessly evidence-based, GICS will continue to nudge governments, NGOs and universities towards the same goal. The task is both ethical and economical; investing in children’s surgery is an investment in the next generation. As the movement for universal health coverage gathers pace, GICS stands at its forefront, working so that the right to health becomes real for every child, everywhere. Each success—whether a district hospital acquiring child-sized anesthetic circuits or a country adding pediatric trauma targets to its health strategy—translates into lives saved and futures restored. Other fields have begun to copy the model: global anesthesia and obstetric-surgery initiatives now cite GICS as proof that equitable collaboration works.

## Data Availability

Data sharing not applicable as no datasets generated and/or analyzed for this study.
